# How and Why Should We Decolonize Global Health Education and Research?

**DOI:** 10.5334/aogh.3787

**Published:** 2022-05-17

**Authors:** Ipek Demir

**Affiliations:** 1 Centre for Ethnicity and Racism (CERS), University of Leeds, United Kingdom

Global health research and education curricula should be decolonized. The “global” in global health helps question ethnocentrism and account for heterogeneity across borders and cultures. It prioritizes the generation and cultivation of different skills, knowledges, practices, and the agility required to deal with a diverse and globalized world and its health. It recognizes that even everyday concepts such as “healthy” or “pain” or “drugs” can be defined and acted upon differently across the globe. Knowing and understanding these differences and bringing them into health research and curriculum matter. What about decolonializing approaches? Why should global health education be decolonized as well as being global? Below I unpack what “decolonizing” can mean for global health education and research, and argue that it needs to (1) recognize the legacies of coloniality in health outcomes; (2) address racial inequalities in health without racializing disease; and (3) increase racial literacy in health education and research. Decolonization should be considered not as a loss but as a gain for global health – it has the potential to make research and curricula more rigorous, leading to better health outcomes.

I define decolonized global health education and research as that which goes beyond valorizing diversity and is able to think through the consequences of the facts of difference and diversity, including racialized attitudes. It takes racism and ethnic injustices seriously, and makes sufficient and explicit references and adjustments in its theorization and practices of health to ethnicity, racism, and global interconnectedness. Hence, I argue that whilst the “global” questions ethnocentrism, the “decolonial” lens makes us aware of, and considers, racial inequalities and injustices to do with health. Below I outline what I see as three core issues that decolonized education and research approaches in global health need to consider.

First, it is necessary to identify and address the lasting legacy of colonial structures, inherited prejudices, and blind-spots within health curricula and research, for example by recognizing that the racial categories used today emerge from 19^th^ century race science. The modern world is largely shaped by colonialism, empires, slavery, and racialized (that is differentiated according to race) hierarchies [1]. Even though colonial domination and empires were officially defeated, coloniality continues: the specific social and economic structures through which racialized differences were constructed were codified into modernity, modern states, economic, social, intellectual and international institutions. As such, decolonial perspectives in health should help uncover and understand differential health outcomes across the world not merely in terms of geographical or regional differences, but also having arisen within a context which is significantly shaped by colonialism, empires and their legacies.

Colonialism not only incorporated a core set of social, cultural, political, and economic hierarchies but also intellectual hierarchies. Societies and education systems have been shaped by this history of the modern world. As such it is important to recognize that curricula and canons are social products in need of revision, just like research programs. Research and education in the Global South are not exempt from these biases either: their curricula can involve their own epistemological biases or carry over prejudices often brought from the Global North. Health education needs to interrogate colonial medical history and race science, identify strategic gaps in curricula, and challenge racialized canons. For example, there is an important need to identify, reveal, and challenge scientific racism, its remnants, and new manifestations (e.g., racialized gene therapy). Research shows lay people as well as medical students in the US endorse beliefs dating back to slavery and colonialism about “Blacks feeling less pain,” “Blacks’ skin being thicker” and that these are related to how doctors undertreat Black Americans as compared to White Americans for pain [2].

Second, decolonizing means that racial divisions and their impact on health should be understood structurally without “racialization of disease.” In social sciences, race is studied *because* there is racism. Race is a contested concept, but has long been accepted as socially constructed, understood as an arbitrary selection of visual characteristics to which society has invested value and importance, with little biological meaning. As the editors of Nature Biotechnology argued in 2005, “[p]ooling people in race silos is akin to zoologists grouping raccoons, tigers and okapis on the basis that they are all stripey.” Yet, race, like other social constructs, for example money, continues to have a real and significant impact on people’s lives. Health education and health research need to thus focus on structural racism – racism is a system which ensures an unequal distribution of resources and penalties between racial groups. Health outcomes are shaped by this, but health interventions can also reproduce racialized outcomes and poor health advice if uninterrupted. For example, despite being known as a “Black illness,” sickle cell disease is not race-bound but related to areas where malaria is dominant; in fact the highest rates in the world are located in India [3].

White, Asian, Latino, and Black are racial categories born in colonialism and from 19^th^ century race science. These racial group categories are themselves hugely varied, socially and historically defined, and since they maintain problematic conceptions of human variation, can lead to essentialism and stereotypes and thus to poor science [4]. Additionally, as racial categories are social, they can vary within and between different countries (e.g., Arabs in the US census are categorized under White, but not in the UK), making the results of global comparisons highly dubious, and can potentially lead to poor clinical judgements. There is therefore the risk that taking race seriously in health sciences can end up reifying race as a biological category, and produce what is referred to as the “racialization of disease,” namely seeing diseases as being inherent to the bodies and genes of racialized groups. Instead, research and education on health need to acknowledge how it is the organization of society which is problematic, giving rise to racialized health outcomes, and based on “racial valuation of disease” [5].

Third, decolonizing global health means that we develop racially literate health research and curricula. Racial literacy here means that racial inequalities are regarded as one of the core social determinants of health [6]. This has become all the more obvious and better known recently as a result of Covid-19 as it disproportionally affected ethnic minorities. Across the Global North diverse, but nearly always racially minoritized populations, including indigenous, Latinx, African Americans, and South Asians, were disproportionally affected not just in terms of infection, but also in terms of deaths [7]. Structural racism intensifies health inequalities. Recent research shows that in the UK despite healthcare being free and universal, Black women have an almost five-fold higher mortality rate in childbirth (in the US it is 3–4 times higher) compared with White women [8], and South Asians receive less potentially beneficial treatments for dementia [9]. These call for health education and practitioners to address such issues, and other prejudices such as in sampling, e.g., the overrepresentation of Whites in medical trials and genomic research. In summary, it has long been known that racism affects the health of minorities through, for example, poor opportunities, mobility, living conditions, differential access to resources and care, racial bias by medical professionals, discrimination, and feelings of inferiority [5,10,11,12]. Such issues need to become mainstream concerns for health research and education and not remain in the margins.

Above I outlined three core issues, issues which are indicative but not exhaustive, that a decolonized global health program could start prioritizing. Paying attention to these can help make health research and teaching more robust, accurate, and interconnected, and also produce better health outcomes for populations. Such interconnectedness also requires interdisciplinarity, including learning from the many years of scholarship in social sciences on race and racism. Last, but not least, often the onus to decolonize curricula and research falls on the shoulders of those minoritized just as gendering curricula has typically fallen on female staff members’ “to do list.” Instead, decolonizing should be taken seriously by everyone, as not to do so makes poor sense not just in education and research but also in health outcomes.
